# Effect of Storage in Distilled Water for Three Months on the Antimicrobial Properties of Poly(methyl methacrylate) Denture Base Material Doped with Inorganic Filler

**DOI:** 10.3390/ma9050328

**Published:** 2016-04-29

**Authors:** Grzegorz Chladek, Katarzyna Basa, Anna Mertas, Wojciech Pakieła, Jarosław Żmudzki, Elżbieta Bobela, Wojciech Król

**Affiliations:** 1 Faculty of Mechanical Engineering, Institute of Engineering Materials and Biomaterials, Silesian University of Technology, ul. Konarskiego 18a, Gliwice 44-100, Poland; Katarzyna.Basa@polsl.pl (K.B.); Wojciech.Pakiela@polsl.pl (W.P.); Jaroslaw.Zmudzki@polsl.pl (J.Ż.); 2 Chair and Department of Microbiology and Immunology, School of Medicine with the Division of Dentistry in Zabrze, Medical University of Silesia in Katowice, Jordana 19, Zabrze 41-808, Poland; amertas@sum.edu.pl (A.M.); ebobela@sum.edu.pl (E.B.); wkrol@sum.edu.pl (W.K.)

**Keywords:** denture base material, antibacterial properties, antifungal properties, aging, antimicrobial filler, silver

## Abstract

The colonization of poly(methyl methacrylate) (PMMA) denture base materials by pathogenic microorganisms is a major problem associated with the use of prostheses, and the incorporation of antimicrobial fillers is a method of improving the antimicrobial properties of these materials. Numerous studies have demonstrated the initial *in vitro* antimicrobial effectiveness of this type of material; however, reports demonstrating the stability of these fillers over longer periods are not available. In this study, silver sodium hydrogen zirconium phosphate was introduced into the powder component of a PMMA denture base material at concentrations of 0.25%, 0.5%, 1%, 2%, 4%, and 8% (*w*/*w*). The survival rates of the gram-positive bacterium *Staphylococcus aureus*, gram-negative bacterium *Escherichia coli* and yeast-type fungus *Candida albicans* were established after fungal or bacterial suspensions were incubated with samples that had been previously stored in distilled water. Storage over a three-month period led to the progressive reduction of the initial antimicrobial properties. The results of this study suggest that additional microbiological tests should be conducted for materials that are treated with antimicrobial fillers and intended for long-term use. Future long-term studies of the migration of silver ions from the polymer matrix and the influence of different media on this ion emission are required.

## 1. Introduction

Yeast-type fungi, including *Candida albicans* (*C. albicans*), can be isolated from the oral cavity of 30%–45% of healthy adults [1,2,3]. In general, fungi are normal commensal organisms within the oral cavity and do not cause problems; however, overgrowth can lead to complications [1]. Wearing dentures produces a micro-environment with low oxygen, low pH, high humidity, elevated temperature, and reduced opportunities for mucosal self-cleaning by saliva, and these conditions are favorable for the growth of microorganisms [1,4]. Thus, yeast can be isolated from the mouths of 50%–65% of removable denture wearers [1,2,3]. The specific conditions in the oral cavity associated with a newly available surface created by denture insertion leads to the rapid formation of denture plaque, which consists of gram-positive streptococci (approximately 40%), gram-positive rods (33%), gram-negative bacteria (approximately 10%), *Staphylococcus aureus* (approximately 6%), and yeast-type fungal colonies [4]. The presence of denture plaque and yeast-type fungi in the oral cavity promotes the formation of denture-induced stomatitis [4,5], which is an inflammatory reaction of the mucosa under dentures. Stomatitis affects approximately 50%–65% of denture wearers [6,7] and has a multifactorial etiology; however, the presence of *C. albicans* has been reported as the primary etiologic cause [8,9]. In addition, in nearly 90% of stomatitis cases, yeast-type fungal species have been isolated [10]. Van Reenen [11] reported that although gram-positive bacteria were more frequently isolated from the mucosa of patients with stomatitis, specific organisms were not associated with lesions, which suggests that the lesions were caused by a community of pathogens. Nyquist [12] reported that the number of bacteria is specific to the individual; however, when dentures are worn for longer periods, bacteria generally increase in number.

Popular chemical denture cleansers have only a limited effectiveness in biofilm removal [13] and can reduce both the mechanical properties [14,15] and color stability [14,15,16] of dentures. Antibiotic therapies for the treatment of fungal infections do not always produce positive results [17], and additional concerns have arisen related to drug resistance among *Candida* spp. and other microorganisms that have been reported over the last decade [18]. *C. albicans* blastospores can penetrate hard poly(methyl methacrylate) (PMMA)-based materials [19]; thus, to avoid complications related to dentures and the associated growth of microorganisms, denture base materials that can damage or strongly resist pathogenic bacteria and fungi are required [20].

Different fillers have been used experimentally to increase the antimicrobial resistance of PMMA denture base materials. Certain metal nanoparticles [20,21,22,23] have been investigated as effective antimicrobial agents, and ceramic particles can provide enhanced resistance against bacteria and yeast [24,25]. Several studies have confirmed the antimicrobial effectiveness of silver nanoparticles incorporated into PMMA denture base materials; however, nanosilver may cause significant color changes, which represents a strong aesthetic limitation in practice. In addition, the long-term stability of the antimicrobial effects has not been examined. In this study, the antimicrobial effectiveness of composites filled with silver sodium hydrogen zirconium phosphate was investigated. Because the number of silver ions released into the environment decreases over time [26], the aim of the work was to investigate the antimicrobial effectiveness of these fillers over three months of storage in distilled water. Our hypothesis was that composites filled with silver sodium hydrogen zirconium would show decreasing antimicrobial efficacy over time.

## 2. Results

### 2.1. Scanning Electron Microscopy (SEM) Investigations

**Figure 1:**
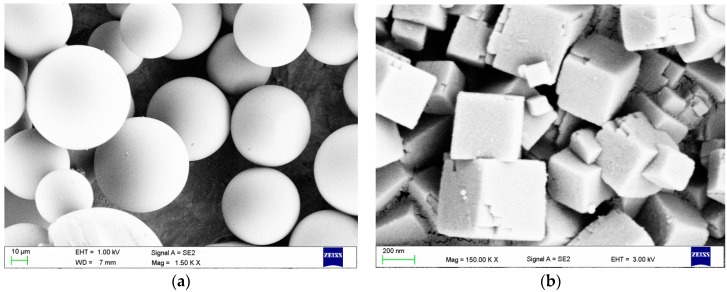
Representative scanning electron microscopy (SEM) images presenting the morphologies of the (**a**) poly(methyl methacrylate) (PMMA) powder; and (**b**) silver sodium hydrogen zirconium phosphate particles.

**Figure 2:**
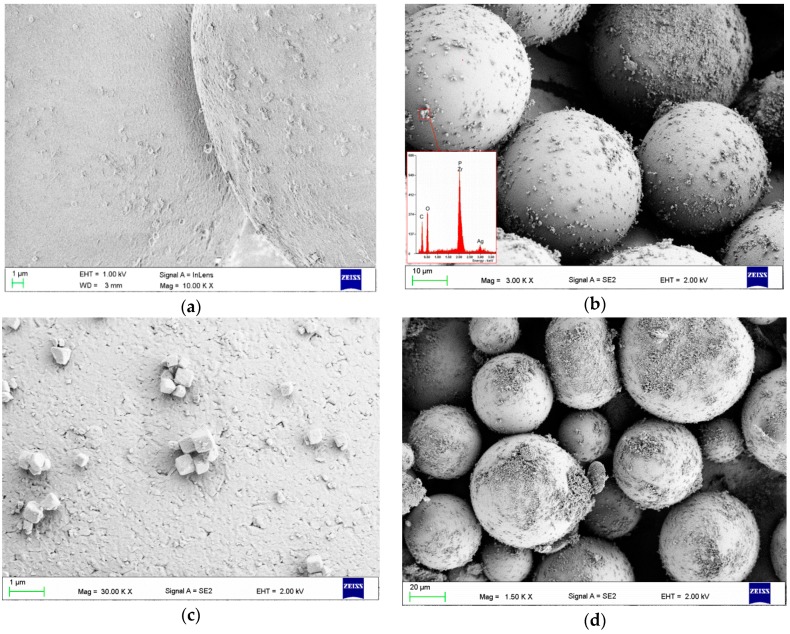
Representative SEM images presenting the surface of the PMMA spheres after filler introduction: (**a**) sphere surface after milling with 0.25% filler; (**b**) surfaces of spheres with 2% filler and the corresponding energy-dispersive X-ray spectroscopy (EDS) spectrum, which confirmed the presence of zirconium, phosphorus and silver; (**c**) smaller aggregates of filler particles; (**d**) spheres covered to a large extent by filler particles when 4% filler was introduced.

SEM images illustrating the morphology of a PMMA powder and filler are presented in Figure 1. The qualitative SEM examinations showed that using a milling time longer than 5 min damaged, (e.g., chipped) the PMMA pearls. SEM images of the PMMA spheres with filler particles on their surface after 5 min of milling are presented in Figure 2. The characteristically cube-shaped filler particles were visible on the surfaces of the PMMA spheres. In addition, above a concentration of 2%, the filler covered the surfaces of certain spheres (Figure 2b), although there were also areas with single particles or smaller aggregations (Figure 2b,c). The areas covered by filler increased along with increasing filler concentration.

**Figure 3:**
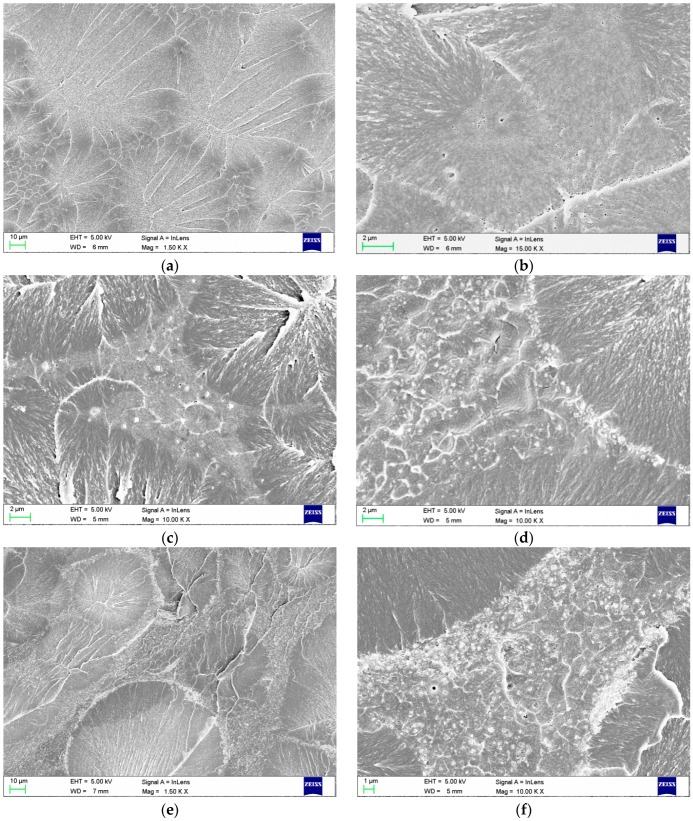
SEM images presenting the morphologies of the fractured samples of polymerized (**a**,**b**) PMMA resin and composites with filler concentrations of (**c**) 1%; (**d**) 2%; (**e**) 4%; and (**f**) 8%.

The morphologies after polymerization are presented in Figure 3. For the resin samples without filler, the areas of pre-polymerized PMMA spheres and areas of PMMA polymerized during sample preparation were clearly visible (Figure 3a). After polymerization, the filler particles were distributed only in areas between the spheres, as expected. At a concentration of 1%, the filler was generally well distributed in the PMMA matrix between the spheres (Figure 3c). Starting at a concentration of 2%, there was an increasing tendency for increased aggregation size, especially on the borders of the pre-polymerized spheres, and PMMA polymerization during sample preparation was observed.

### 2.2. Microbiological Tests

**Figure 4:**
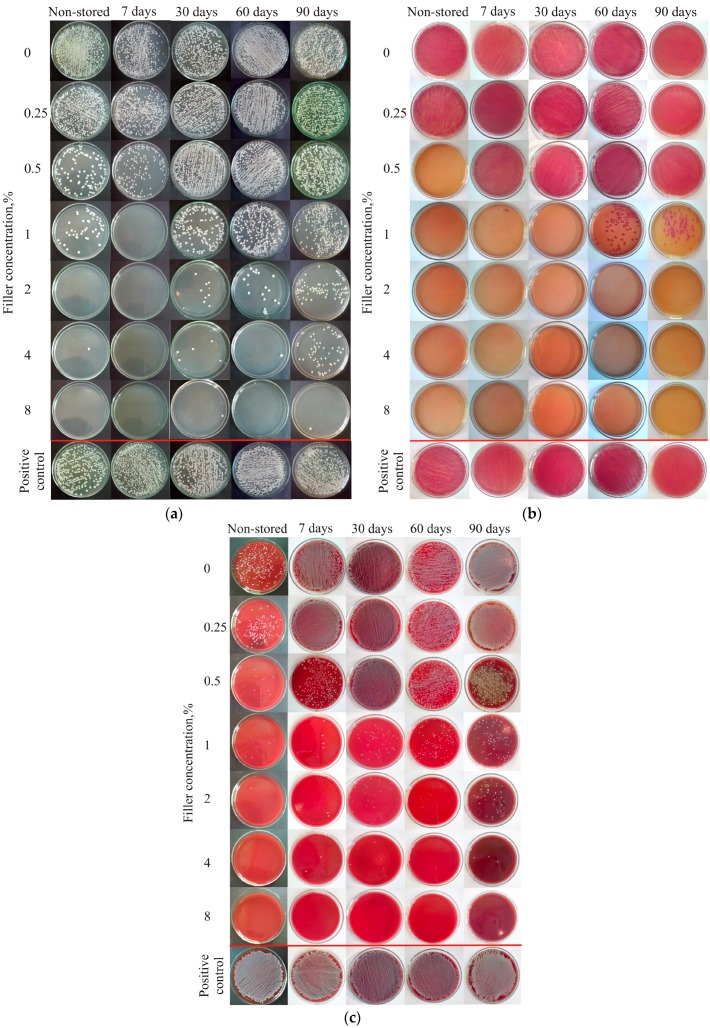
Representative results of the antifungal tests against (**a**) *Candida albicans* ATCC 10231; (**b**) *Escherichia coli* ATCC 25922; and (**c**) *Staphylococcus aureus* ATCC 25923 after 17 h of incubation with samples of the PMMA resin and composites previously stored in distilled water.

The results of the antifungal and antibacterial tests are presented in Figure 4, and the *C. albicans* survival rate (SR) values are listed in Table 1. For different storage durations, increasing the filler concentration had a significant effect on the SR of the fungi (Table 1). For PMMA without filler, antifungal effects were not observed before or after storage. Storage duration had a significant influence (*p* < 0.05) on the SR of the bacteria for particular filler concentrations starting from 0.5%, although after 30 days of storage, the SR of fungi at a concentration of 0.5% was 100%. For a filler concentration of 1%, the SR remained stable below 0.01% over 60 days, whereas the median of SR increased to 57.21% after 90 days.

Although increases in the SR were observed after 90 days of storage with filler concentrations of 2% and 4% introduced to the PMMA resin, these increases were smaller than those observed for lower filler concentrations. For the highest filler concentration, the SR was generally similar over 3 months of storage, whereas the SR increased from below 0.01% after 60 days to 0.17% after 90 days. For filler concentrations from 2% to 8%, the SR values were 0% after 30 days, and only a few fungal colonies survived after prolonged storage.

The *E. coli* SR values are listed in Table 2. For different storage durations, increases in filler concentration had a significant effect (*p* < 0.05) on the SR of the bacteria (Table 2). For PMMA without filler, an antimicrobial effect was not observed before or after storage. Storage duration had a significant influence (*p* < 0.05) on the SR of the bacteria for filler concentrations of 0.25% to 1% in the PMMA resin. Starting from a filler concentration of 2%, an antimicrobial effect was also noted, although no significant effects (*p* > 0.05) on the SR were observed over the 3-month experiment. For a filler concentration of 2%, only a few bacterial colonies survived after 90 days of storage.

The *S. aureus* SR values are listed in Table 3. For different storage durations, increases in the filler concentration had a significant effect (*p* < 0.05) on the SR. The storage duration had a significant influence (*p* < 0.05) on the SR of the bacteria for all the tested filler concentrations except 8% (*p* = 0.637). The initial antibacterial effects of all the materials are noted. After the first seven days of storage, the number of viable *S. aureus* colonies increased, and the SR was below 0.01% at filler concentrations of 0.5% and above. After 30 days, reduced SR values were noted, starting from a filler concentration of 1%. Beginning at a concentration of 1%, the median of SR values were below 0.01% for all of the materials and storage durations, although small changes in the number of viable *S. aureus* colonies were still observed.

## 3. Discussion

The use of antimicrobial fillers is a common method of enhancing the antimicrobial properties of PMMA denture base materials, and several investigations that have tested antimicrobial fillers *in vitro* have reported increased material resistance against fungi and bacteria [27]. Commercially available, two-component, powder–liquid systems are typically used as a matrix in these types of investigations, and because of practical and technological reasons, such a system was also used in this study. Two methods of incorporating antimicrobial fillers into PMMA denture base materials are commonly reported*—i.e.*, fillers can be mixed into either the liquid component [21,28,29] or the powder component [30,31,32]. Both methods allow the filler particles to be located between the pre-polymerized spheres after sample polymerization, which reduces the polymerization-induced shrinkage of the material. In the current investigation, the filler was mixed with the powder component in a ball mill. The filler was not introduced into the liquid component because the large differences in density between the silver sodium hydrogen zirconium phosphate (2914 kg/m^3^) [33] and the MMA-based liquid (950 kg/m^3^) would have increased the risk of sedimentation during component storage prior to sample polymerization.

The distribution of filler particles and their aggregation between spheres is visible in Figure 2. Such a distribution is a technological limitation and has not been addressed in related studies of nanofiller and microfiller incorporation into commercially available PMMA denture base materials. This problem can be resolved by introducing additional filler particles during the suspension polymerization process that produces the PMMA spheres. At the laboratory scale, this approach requires additional investigation for the development of novel materials with suitable properties for use as denture base materials. Therefore, when testing new fillers for PMMA denture base materials, it is common practice to use commercially available products in the first process step. Examples of the process of manufacturing pre-polymerized spheres during or before filler introduction have only been presented by Acosta-Torres *et al.* [21,34]. In our study, the milling process did not allow the production of a homogenous filler distribution either across the surface of the spheres before polymerization or between the spheres after polymerization. These inhomogeneities, which were confirmed by SEM investigations, were greater with higher filler concentrations. The problems associated with filler aggregation in the polymeric matrix have been well-documented by laboratory-scale investigations [27], and such issues may affect the antimicrobial effectiveness and reduce the mechanical properties of the material. The large aggregations, such as those observed with higher filler contents near the borders of the pre-polymerized PMMA spheres, can act as stress concentrators, thus reducing mechanical properties such as flexural and impact strength. In addition, future investigations should examine the influence of filler introduction on the sorption, solubility, and residual monomer of the material.

Reducing discoloration caused by additives such as nanosilver was one of the reasons why a white filler was used in this study. PMMA resin without pigments was used in previous investigations, and we observed increased white coloration and reduced transparency with increasing filler concentrations. When pigmented PMMA materials were used experimentally, the color changes were much less apparent; however, the impact on transparency was approximately the same as previously observed. These unfavorable changes are typical and are associated with the use of nano- and microfillers. The additives did not affect the taste or odor of the materials.

The morphology of the material with pre-polymerized spheres also influenced the choice of the method used in the microbiological tests. The powder–liquid ratio recommended by the manufacturer was 3.5 g/1.4 mL; therefore, the filler in the samples was dispersed within a sample mass of approximately 28%, which created areas that were polymerized from the liquid component between spheres. The local filler concentrations in the areas between pre-polymerized spheres for different initial filler concentrations mixed with PMMA powder were 0.6%, 1.2%, 2.4%, 4.8%, 9.7%, and 19.4% respectively (*w*/*w*). For the total sample mass, the filler concentrations were 0.18%, 0.36%, 0.72%, 1.45%, 2.9%, and 5.8% (*w*/*w*). Therefore, for the microbiological assays, the samples were incubated with microorganism suspensions, and the emission of silver ions into the medium determined the SR of the tested microorganisms (*i.e.*, fungi or bacteria). The comparative effectiveness of the studied materials has yet to be determined using different microbiological tests, such as live/dead cell viability assays [35], where the surface colonization of the samples is analyzed using fluorescence microscopy because the filler-free areas may potentially be colonized. However, for samples with low microorganism SR values in the environment, the viability of microorganisms on the material surface should also be very low. Furthermore, although the local environment in the oral cavity under dentures is wet, the reduced self-cleaning by saliva [1] could allow the number of silver ions present to increase with increasing time wearing the denture. It is important to consider that the reduced mucosal self-cleaning by saliva under dentures is a significant factor that favors the growth of fungi and bacteria [1,4]. To confirm these assumptions, microbiological tests should be performed to investigate the adherence of bacteria on sample surfaces, which should be more representative of denture surface areas that do not have continuous and direct contact with the mucosa and are cleaned by saliva during denture wearing. A non-homogenous distribution of antimicrobial fillers should also be considered with respect to the cytotoxicity of the obtained materials. Indeed, the cytotoxic potential of different antimicrobial agents has been reported [36,37,38,39,40], and the concentrations required to enhance the antimicrobial resistance of modified denture base materials have been reported to be both non-cytotoxic and non-genotoxic [21,34,41]. The concentration of silver in the filler was 10%; thus, the concentration of silver in the investigated materials was not greater than 80 ppm in the total volume. Toxicological data [33] have shown that the composites used in our study should not present cytotoxic risks, but these low values can be locally enhanced because of the noted inhomogeneities caused by using pre-polymerized spheres. Silver ions have been reported to cause material toxicity [27]. In addition, studying the migration of silver ions from fillers in a polymer matrix can be combined with abrasion studies. When dentures are worn, more silver could be released from the polymer matrix through contact with the mucosa or while chewing, thereby improving antimicrobial activity. In contrast, selective abrasion of the antimicrobial filler from the matrix may lead to an accelerated loss of the ability to release silver ions into the environment. Another remaining question is the influence of these suggested abrasion effects on the toxic potential of the filler. For these reasons, the release of ions from and the cytotoxic potential of proposed materials must be extensively investigated over long periods.

The effect of storing samples in distilled water on the SR of microorganisms was also investigated in our study. Numerous studies have investigated the initial antimicrobial properties of denture base materials with different additives [27]. Pereira-Cenci *et al.* [42] showed that the delivery of new dentures manufactured from commercially available materials significantly decreased the number of *Candida* species after the first month of wear; however, the *Candida* levels increased over the following month. This result shows that the regular replacement of dentures may not prevent yeast colonization. The overall conclusion from these studies is that the most important aspect of new antimicrobial denture base materials is the stability of the resulting efficacy. In our study of a commercially available material, antifungal and antibacterial effects were not observed, which corresponds with investigations that have shown a lack of resistance to *C. albicans* colonization [22,27]. In our investigations of *S. aureus,* the SR decreased for samples that were not stored in distilled water. This unexpected result was confirmed by duplicating the tests, and it may have been related to the release of residual leachable components into the environment. Moreover, similar results have been reported for other dental materials [19,42,43,44]. Denture base resins contain polymerizable methylacrylate/acrylate monomers and methacrylate copolymers. The antimicrobial activity of certain acrylic and methacrylic copolymers has been demonstrated [45,46]. The manufacturer that supplied the resin used in this study recommends storing new dentures for at least 12 h in tepid water prior to use to reduce the concentration of rinsable or soluble components; however, this was not performed for the non-stored samples. This step of the experimental protocol appeared to have an effect on the findings because a reduction in the surviving number of bacteria was not confirmed for the samples stored in distilled water. Similar antibacterial activity for the non-stored and non-filled samples was not observed for *E. coli*, although Farah *et al.* [47] and Lu *et al.* [48] investigated the use of polymeric materials and reported significantly higher antibacterial activity against *S. aureus* than for *E. coli*. Several studies have reported that for antimicrobial additives and polymers, the effect against *S. aureus* was lower than that against *E. coli* [45,49,50], a difference that indicates significant variability in the results.

In our study, a *C. albicans* strain was used as a representative typical pathogenic microorganism associated with denture wearing [4]. The *C. albicans* SR increased for samples after longer storage periods. Although the composites with 0.25% filler initially showed low antifungal properties, these properties were generally lost after the first week. Strong antifungal properties after two months of storage were confirmed with filler concentrations of 1% or greater. After three months, the composites with a filler concentration of 1% to 4% showed an increase in SR, although the value was still relatively low. When analyzing SR results, the surviving number of fungi in CFU/mL (Table 2) should be considered, because small differences in the SR may result from methodological limitations.

*E. coli* and *S. aureus* are not typical pathogenic microorganisms associated with denture wearing; therefore, they were used as representative strains of gram-positive and gram-negative bacteria. The results for these bacterial strains showed that after one week of storage, the samples with filler concentrations of 0.25% and 0.5% lost their antibacterial activity, whereas the samples with the largest filler concentrations presented low SR values (*i.e.*, below 0.01%). Even when there were no changes or only small changes in the SR, the surviving number of bacteria and fungi in CFU/mL slightly increased over time. Prolonged storage may lead to further increases in SR and reductions in antimicrobial properties; this would be consistent with other results obtained for dental materials, in which the emission of antimicrobial Pt or Ag ions decreased strongly during the first hour of storage [20,26]. In addition, Nam *et al.* [23] and Sokołowski *et al.* [26,51] showed that ion emissions reduced with increasing storage duration. Kampmann *et al.* [52] reported that the antimicrobial activity of the used filler was activated under humid conditions and that the mechanism of silver ion release was based on the exchange of ions from the wet environment with silver from the inorganic, insoluble carrier. This mechanism could be favorable for PMMA denture base resins due to their water sorption and porosity by reducing the possibility of microorganisms penetrating the material. In contrast, the results obtained by Qin *et al.* [53] for silver sodium hydrogen zirconium phosphate-containing chitosan fibers suggested that in liquid media, such as saliva, the presence of sodium chloride and proteins can increase the number of silver ions released into the environment. This increase could lead to a faster release of silver ions *in vivo* and an earlier loss of antimicrobial activity.

The *in vitro* findings presented here must be confirmed with further experiments conducted under *in vivo* conditions. Because of the numerous additional factors related to *in vivo* conditions, such as the deposition of salivary proteins on the dentures, differentiated specificity of cleaning materials by saliva, direct contact with various foods and liquids and other environmental properties, *in vitro* tests are only a starting point for evaluating the possibility of enhancing the antimicrobial properties of the studied materials.

## 4. Materials and Methods

### 4.1. Material Preparation

As a matrix, the commercially available PMMA heat-cured denture base resin Meliodent Heat Cure (Heraeus Kulzer, Hanau, Germany) was used. The material is a two-component “*powder–liquid*” system. To eliminate the possibility of sedimentation during material storage, the filler was added only to the “powder” component. As an antimicrobial filler, silver sodium hydrogen zirconium phosphate (Milliken Chemical, Spartanburg, SC, USA) was used. The filler was introduced into the PMMA component by mixing with a Pulverisette 5 planetary ball mill (Fritsch, Idar-Oberstein, Germany). During milling, 50 ZrO_2_ balls with a diameter of 10 mm were used. In the first stage, different milling times (1, 3, 5, 10, 30 min) with a frequency of rotation of 400 rpm were used to establish the milling parameters. Experimental millings were performed with 10 g of material sample and 3% (*w*/*w*) filler. To determine the best parameters, two evaluation criteria were established: the uniform distribution of filler particles on the PMMA spheres and a lack of visible damage to the PMMA spheres. For the obtained powder–filler compositions, samples were collected from randomly selected locations, placed on carbon tape (Agar Scientific, UK) and qualitatively examined with a Zeiss SUPRA 35 (Carl Zeiss, Konstanz, Germany) scanning electron microscope at accelerating voltages of 1 and 5 kV. The best results were obtained at a milling time of 5 min, and this condition was used for all of the material preparations. For the following investigations, powder–filler compositions with filler concentrations of 0.25%, 0.5%, 1%, 2%, 4%, and 8% (*w*/*w*) were produced.

### 4.2. Preparation of Polymerized Samples

Plates measuring 65 mm in length, 45 mm in width, and 2.5 mm in thickness were used to prepare samples for microbiological tests, and SEM investigations were performed using a standard flasking procedure used in prosthetics. The materials were polymerized in accordance with the instructions of the resin manufacturer.

The polymerized plates were preliminarily wet-ground using 220-grit abrasive papers (Struers A/S, Copenhagen, Denmark) to eliminate any possible unevenness and to initially standardize the thickness. After this grinding process, the thickness of the plates was 2.2 ± 1 mm. Square pieces measuring 12 ± 1 mm on a side were cut from the plates for the microbiological test, and rectangular pieces measuring 10 ± 1 × 20 ± 1 mm were cut for the SEM investigation. The edges and surfaces of the samples were wet-ground using 220-grit abrasive paper to remove imperfections on the edges after cutting (all samples) and to standardize the dimensions (only samples for the microbiological tests).

The samples for the microbiological tests were then rinsed and wet-ground using 500-grit abrasive paper to remove the scratches made by the previous grinding process; these samples were then rinsed again. The final samples were 10.0 ± 0.2 mm on a side and 2.0 ± 0.2 mm thick. Next, the samples were stored in distilled water at 37 ± 1 °C for 7 days ± 2 h, 30 days ± 2 h, 60 days ± 2 h or 90 days ± 2 h. Each group of samples (*i.e.*, material type–storage duration–microorganism strain) was placed separately into 250 ± 10 mL of distilled water in a glass crystallizer and covered with a petri dish. The crystallizers were placed in stainless steel chambers that contained distilled water and were equipped with a heater, a pump for water motion, and a temperature controller. The water in the crystallizers and chambers was replaced weekly. After being stored, the samples were placed inside desiccators containing freshly dried silica gel and were dried at 37 ± 1 °C for 4 h. An additional sample group was not stored in distilled water. All samples were packed for plasma sterilization.

### 4.3. SEM Investigations of Polymerized Samples

Samples for the SEM investigations after wet-grinding were placed inside desiccators containing freshly dried silica gel and were then dried at 40 ± 1 °C for 2 h; then, a freeze-fracturing process was used. The samples were immersed in liquid nitrogen, broken, etched with an 85% (*w*/*w*) solution of orthophosphoric acid (Avantor, Gliwice, Poland) for 15 s, and then sputtered with silver. The obtained fractures were observed by SEM using a Zeiss SUPRA 35 scanning electron microscope at accelerating voltages of 1 and 5 kV.

### 4.4. Microbiological Tests

The *in vitro* antimicrobial activities of the composites were examined according to the methods described by Melaiye *et al.* [54] and Xu *et al.* [55], with certain modifications. The following standard strains of microorganisms were used: gram-positive *Staphylococcus aureus* ATCC 25923 (*S. aureus*), gram-negative *Escherichia coli* ATCC 25922 (*E. coli*) and the yeast-type fungus *Candida albicans* ATCC 10231 (*C. albicans*). These standard microorganism strains were acquired from the American Type Culture Collection (ATCC). Polymerized and sterilized samples of the studied composites were introduced individually in 2 mL of fungal or bacterial suspensions in tryptone water, which contained approximately 1.5 × 10^5^ CFU/mL (CFU—*colony forming units*) of *C. albicans*, *E. coli*, or *S. aureus*. Suspensions of 1.5 × 10^5^ CFU/mL of fungi or bacteria in tryptone water were tested as a positive control. Pure tryptone water was tested as a negative control. All of the samples with microorganism suspensions were incubated in a shaking incubator for 17 h at 37 °C for *E. coli* and *S. aureus* and at 35 °C for *C. albicans*. After incubation, 20 μL of each suspension was seeded onto Sabouraud agar plates for *C. albicans,* Columbia agar with 5% sheep blood plates for *S. aureus* and MacConkey agar plates for *E. coli*. The Sabouraud agar, Columbia agar, and MacConkey agar were purchased from bioMerieux (Marcy l’Etoille, France). The cultured plates were incubated at 37 °C for 24 h (bacteria) or 35 °C for 48 h (yeast). Then, the number of bacterial or fungal colonies (CFU) were counted. These counts were used to calculate the surviving number of bacteria or fungi [56]. Each material–storing condition with each standard strain of microorganisms was tested in quadruplicate.

The SR was calculated according to the following equation:

(1) $$\mathrm{SR}=\frac{V_{t}}{V_{c}}\times 100\%,$$

where SR is the survival rate (%), *V*_c_ is the number of viable fungal or bacterial colonies of the positive control, and *V*_t_ is the number of viable fungal colonies of the test specimen.

### 4.5. Statistical Analysis

The results were subjected to a statistical analysis using Statistica software (version 10, StatSoft, Tulsa, OK, USA), and the non-parametric Kruskal-Wallis test (α = 0.05) was also used.

## 5. Conclusions

The PMMA denture base material was successfully modified with ceramic antimicrobial filler, and the obtained composites showed a low initial SR for the tested fungi and bacteria. The hypothesis that the composites filled with silver sodium hydrogen zirconium would show decreased antimicrobial efficacy over time was confirmed. Within the limits of this study, the following conclusions were formulated.

- The increased SRs with prolonged storage duration indicate that further storage would result in a loss of resistance against microorganisms. For materials modified with antimicrobial fillers, additional long-term storage tests should be conducted. For denture base materials (and other similarly used materials), it is important to consider that new dentures may not be colonized immediately and that the initial resistance against fungi and bacteria should only be used as a starting point.
- The use of commercially available, two-component, PMMA denture base materials presents limitations because the pre-polymerized spheres are a major cause of inhomogeneity among the obtained composites. This inhomogeneity creates areas free of filler, which affects the properties of the materials. Therefore, additional research should be performed on the introduction of fillers to these spheres during suspension polymerization to obtain better distributions at lower filler concentrations and to improve the antimicrobial properties.
- Long-term studies of the migration of silver ions from the polymer matrix and the influence of different media on ion emission need to be performed. Future results should be coupled with the presented research as well as with cytotoxicity studies.

## Figures and Tables

**Table 1:** Survival rate (SR) of *Candida albicans* ATCC 10231 with the PMMA resin and composites with different filler concentrations stored in distilled water and Kruskal-Wallis test results (α = 0.05).

Filler Concentration %	Survival Rate (SR) of *Candida albicans* ATCC 10231 (%)									
	Non-Stored (*p* = 0.048) ^‡^		7 Days (*p* = 0.003) ^‡^		30 Days (*p* = 0.004) ^‡^		60 Days (*p* = 0.004) ^‡^		90 Days (*p* = 0.002) ^‡^	
	Median	Min/Max	Median	Min/Max	Median	Min/Max	Median	Min/Max	Median	Min/Max
0 (*p* = 0.217) ^†^	107.24	92.35/123.29	96.75	87.11/101.96	100	*	100	*	119.59	99.18/132.78
0.25 (*p* = 0.223) ^†^	57.2	48.89/89.35	100.03	66.06/112.48	100	*	100	*	88.14	85.11/139.3
0.5 (*p* = 0.004) ^†^	5.25	0.92/11.77	24.5	17.07/34.38	100	*	100	*	99.65	64.18/141.4
1 (*p* = 0.042) ^†^	0	0/7.04	0.06	0/0.12	<0.01	<0.01/0.02	<0.01	#	57.21	45.93/68.49
2 (*p* = 0.01) ^†^	0	0/1.84	0	*	<0.01	0/<0.01	<0.01	#	10.29	6.86/30.11
4 (*p* = 0.007) ^†^	0.52	0/1.84	0	*	<0.01	0/<0.01	<0.01	0/<0.01	18.43	8.72/45.69
8 (*p* = 0.035) ^†^	0.23	0/0.92	0	*	<0.01	0/<0.01	<0.01	0/<0.01	0.12	0.12/0.34

*p*-values marked ^†^: refer to the SR differences listed in rows (different storage durations for a particular material); and those marked ^‡^: refer to the SR differences listed in columns (different filler concentrations for a particular storage duration); *: no changes in quadruplicate; #: both were below 0.01 but more than 0.

**Table 2:** Survival rate (SR) of the *Escherichia coli* ATCC 25922 standard strain for the investigated materials stored in distilled water and Kruskal-Wallis test results (α = 0.05).

Filler Concentration %	Survival Rate (SR) of *Escherichia coli* ATCC 25922 (%)									
	Non-Stored (*p* < 0.001) ^‡^		7 Days (*p* < 0.001) ^‡^		30 Days (*p* < 0.001) ^‡^		60 Days (*p* < 0.001) ^‡^		90 Days (*p* < 0.001) ^‡^	
	Median	Min/Max	Median	Min/Max	Median	Min/Max	Median	Min/Max	Median	Min/Max
0 (*p* = 1) ^†^	100	*	100	*	100	*	100	*	100	*
0.25 (*p* < 0.001) ^†^	<0.01	#	100	*	100	*	100	*	100	*
0.5 (*p* < 0.001) ^†^	0	*	100	*	100	*	100	*	100	*
1 (*p* = 0.002) ^†^	0	*	0	*	0	*	<0.01	#	<0.01	#
2 (*p* = 0.406) ^†^	0	*	0	*	0	*	0	*	0	0/<0.01
4 (*p* = 1) ^†^	0	*	0	*	0	*	0	*	0	*
8 (*p* = 1) ^†^	0	*	0	*	0	*	0	*	0	*

*p*-values marked ^†^: refer to the SR differences listed in rows (different storage durations for a particular material); and those marked ^‡^: refer to SR differences listed in columns; *: no changes in quadruplicate; #: both below 0.01 but more than 0.

**Table 3:** Survival rate (SR) of the *Staphylococcus aureus* ATCC 25923 standard strain for the investigated materials stored in distilled water and Kruskal-Wallis test results (α = 0.05).

Filler Concentration %	Survival Rate (SR) of *Staphylococcus aureus* ATCC 25923 (%)									
	Non-Stored (*p* = 0.005) ^‡^		7 Days (*p* = 0.001) ^‡^		30 Days (*p* = 0.001) ^‡^		60 Days (*p* = 0.001) ^‡^		90 Days (*p* = 0.001) ^‡^	
	Median	Min/Max	Median	Min/Max	Median	Min/Max	Median	Min/Max	Median	Min/Max
0 (*p* < 0.001) ^†^	<0.01	#	100	*	107.69	*	100.0	*	106.25	*
0.25 (*p* < 0.001) ^†^	<0.01	#	100	*	107.69	*	100.0	*	106.25	*
0.5 (*p* = 0.001) ^†^	<0.01	0/<0.01	<0.01	#	107.69	*	100.0	*	106.25	*
1 (*p* = 0.003) ^†^	<0.01	#	<0.01	#	<0.01	#	<0.01	#	<0.01	#
2 (*p* = 0.02) ^†^	<0.01	0/<0.01	<0.01	#	<0.01	#	<0.01	#	<0.01	#
4 (*p* = 0.013) ^†^	<0.01	0/<0.01	<0.01	#	<0.01	#	<0.01	#	<0.01	#
8 (*p* = 0.637) ^†^	0	0/<0.01	<0.01	0/<0.01	<0.01	0/< 0.01	<0.01	0/<0.01	<0.01	0/<0.01

*p*-values marked ^†^: refer to the SR differences listed in rows (different storage durations for a particular material); and those marked ^‡^: refer to SR differences listed in columns. *: no changes in quadruplicate; #: both below 0.01 but more than 0.
